# Supplementary material for: Automated analysis of C. elegans behavior by LabGym: an open-source, AI-powered platform
Source: G3 (Bethesda). 2026 May 6;16(7):jkag120. doi: 10.1093/g3journal/jkag120 (PMC13334171; doi:10.1093/g3journal/jkag120)
Supplement: jkag120_Supplementary_Data [file jkag120_supplementary_data.zip › Supplemental_Material_Title_and_Legends_G3-2025-406423.docx]

**Figure S1. Locomotion trajectories of aging worms, color-coded by individual and by time**

For each age, a pair of trajectory images was generated to depict individual worm movement paths, color-coded by individual (left image) or time (right image). For trajectory images color-coded by individual, different colors indicate different individuals. For trajectory images color-coded by time, color gradients indicate temporal progression of movement (earlier timepoints in cool tones, later timepoints in warm tones). These visualizations highlight age-dependent changes in the activity patterns and movement trajectories of worms across adult lifespan.

**Table S1. Summary Table of trained Categorizer metrics**

Table summarizing the metrics of the trained Categorizer (overall accuracy, Animation Analyzer, Pattern Recognizer, and every behavior category’s precision, recall, and f1-score).

**Video S1. Representative behavior examples used for Categorizer training**

Representative behavior examples used to train the Categorizer. The sorting criteria and total number of example pairs for training each behavior category are detailed in Methods.

**Video S2. Annotated videos of N2 worm locomotion by *LabGym***

**Left:** Behavioral video of six Day 1 adult N2 worms with frame-by-frame annotation of computed behavior category and associated probability. 5x speed, 20 frames per second.

**Right:** Behavioral video of six Day 1 adult N2 worms with frame-by-frame annotation of computed behavior category and associated probability. 5x speed, 20 frames per second.

**Video S3. *LabGym* maintains detection accuracy during multi-worm collision**

A video example of a multi-worm collision event where *LabGym* maintains analysis accuracy before, during, and after the collision of two forward crawling worms without identity switching. Although *LabGym* is capable of differentiating individual worms when they physically contact each other in most scenarios, it must be noted that the Categorizer may miscategorize behaviors during and/or a few frames post-collision with an error rate of ~30%, but can typically correct itself relatively quickly following entanglement. Given the Categorizer’s overall accuracy of ~90%, such miscategorizations during collisions account for much of the remaining ~10% inaccuracy. Retraining the Categorizer with more behavior examples involving multi-worm collisions is likely to improve its accuracy.
