# Supplementary figures and images for: Automated analysis of C. elegans behavior by LabGym: an open-source, AI-powered platform
Source: G3 (Bethesda). 2026 May 6;16(7):jkag120. doi: 10.1093/g3journal/jkag120 (PMC13334171; doi:10.1093/g3journal/jkag120)

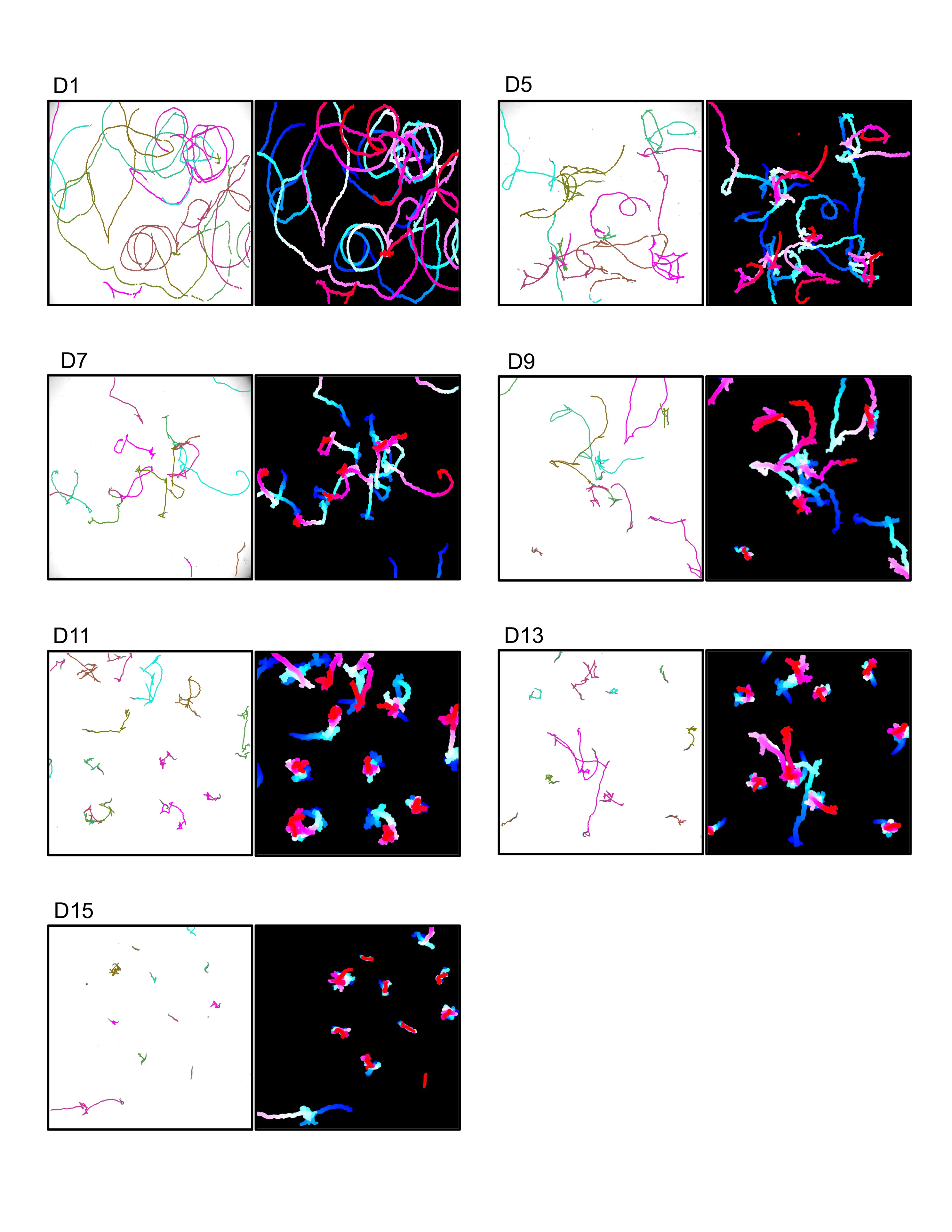

Supplement: jkag120_Supplementary_Data [file jkag120_supplementary_data.zip › Figure_S1_G3-2025-406423.tif]
